# Sealed Duodenal Perforation by nail ingestion in a Child

**DOI:** 10.12669/pjms.343.14331

**Published:** 2018

**Authors:** Fatima Naumeri, Bilal Qayyum, Muhammad Sohaib Yousaf

**Affiliations:** 1 Dr. Fatima Naumeri, MCPS. FCPS. Department of Pediatric Surgery, King Edward Medical University, Lahore, Pakistan; 2 Dr. Bilal Qayyum, Department of Pediatric Surgery, King Edward Medical University, Lahore, Pakistan; 3 Dr. Muhammad Sohaib Yousaf, Department of Pediatric Surgery, King Edward Medical University, Lahore, Pakistan

**Keywords:** Children, Duodenal perforation, Nail ingestion, Foreign body

## Abstract

Unintentional foreign body ingestion is common among children. Normally, these ingested foreign bodies pass spontaneously and the rest can be removed endoscopically; only few ingested foreign bodies lead to complications and need surgical intervention. We are reporting a case of accidental nail ingestion in a 10-year-old child which led to a sealed perforation of duodenum. Operative management included primary duodenal repair after removal of nail. Post operative recovery was smooth and oral was started on day 5. We recommend that all ingested sharp and large foreign bodies should be removed endoscopically, if not passed spontaneously.

## INTRODUCTION

Foreign body ingestion is a potentially serious problem with a peak incidence in children aged six months to three years and involving both sexes.^1^ Majority of the patients who have swallowed foreign bodies remain asymptomatic (90%) and these foreign bodies pass through body spontaneously.^2^ Rest can be removed endoscopically. Serious complications such as bowel perforation, and obstruction can occur in 1% of the cases and these cases require surgical intervention.^1^

Coins are the most commonly swallowed foreign body worldwide, followed by fish bones. Other ingested materials vary according to traditions and nutritional habits of the population.^3^ There are less reported cases of nail ingestion leading to duodenal perforation in children. In this manuscript, we present a case of nail ingestion in a boy of 10 years of age, causing sealed perforation of duodenum.

## CASE REPORT

A 10-year-old boy presented to Pediatric Surgery Department, Mayo Hospital in October 2017 with history of accidental ingestion of metallic nail and epigastric pain for 20 days. Parents consulted a private hospital and was advised observation. The nail did not pass spontaneously and patient started having colicky upper abdominal pain and was brought to our hospital.

There was no previous history of foreign body ingestion and patient’s psychological evaluation was normal. On examination, there was tenderness in epigastric region and right hypochondrium. Rest of the abdominal examination was normal. Plain X-ray abdomen showed a nail in right upper quadrant and its position had not changed from first X-ray (Fig.1A). Laboratory reports was unremarkable.

**Fig.1a:**
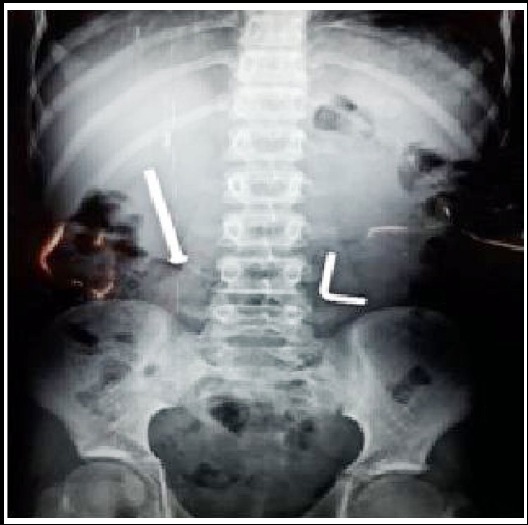
Ingested nail seen on X Ray erect abdomen.

**Fig.1b:**
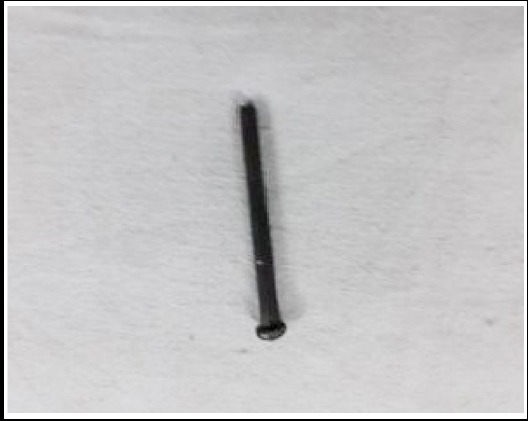
Nail after removal.

After admission and informed consent, emergency exploration was performed. Peroperatively, a metallic nail was found impacted in 2^nd^ part of duodenum. The head was embedded in the secondpart causing a small perforation in the duodenum which was sealed by omentum (Fig.2A). No spillage was noted in the peritoneal cavity. Through same perforation nail was delivered (Fig.2B). Duodenal perforation was repaired primarily after debridement of edges and drain was placed near repair.

**Fig 2a:**
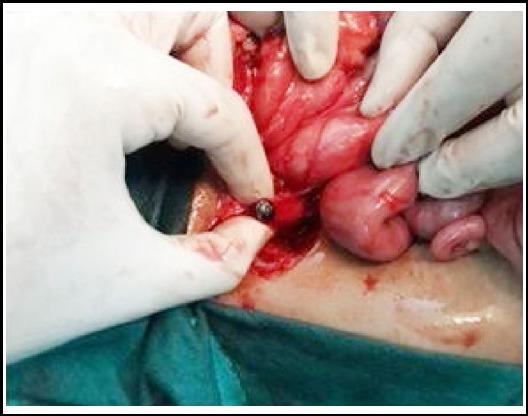
Head of nail embedded in second part of duodenum causing sealed perforation.

**Fig.2b:**
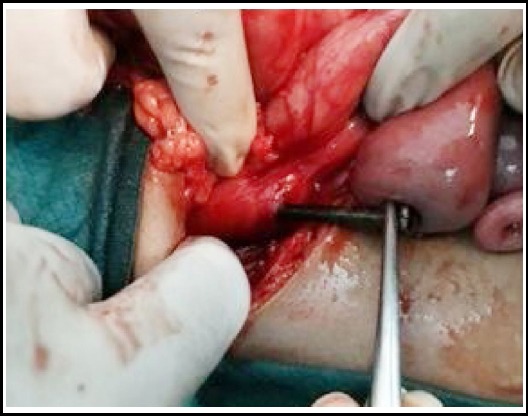
Removal of nail through duodenal perforation.

Postoperative recovery was smooth and uneventful. Drain and nasogastric tube were removed on the fourth postoperative day and oral liquids were started on day 5. Patient was discharged on day 9. On subsequent follow up, he was symptom free and okay. Permission for publication was taken from father and ethical approval taken from institute.

## DISCUSSION

Foreign body ingestion can occur unintentionally in children, adults, elderly and patients with mental retardation.^4^ Intentional foreign body ingestion is most commonly seen in adult patients with intellectual or mental disabilities, significant substance abuse, psychiatric disorders, or external motivations (such as avoidance of a jail sentence). Repeat foreign body swallowing may be part of a syndrome of self-mutilation and/or attention-seeking behavior.^5^

In our ward during the last year alone, 15 cases of foreign body ingestion were admitted. Out of these, three needed operative management, one had a duodeno-cecal fistula due to hairpin, the second had ileal perforation due to battery ingestion and the third one is our case report.

Though common age of presentation is three months to six years for foreign body ingestion in children^1^,^4^,^6^, but in our case, accidental ingestion was noted in a 10-year-old boy and it needed surgical intervention.

Most of the documented cases requiring surgery are due to sharp objects like hairpin, fish bones, and table spoons etc and may present as internal fistulae or fecal fistula, abscess, peritonitis or intestinal obstruction.^2^ There are less reported cases of nail ingestion causing duodenal perforation especially in children; although the rigid nature of the duodenum as well as its deep transverse rugae and sharp angulations make it a common site for the entrapment of long and sharp-ended objects.^7^ Still perforations of duodenum are relatively rare (5-10%) and present in a more innocuous manner.^2^,^8^

Main radiological modality to diagnose the ingested radio opaque foreign body is X-ray. It can depict the change in position of foreign body inside gastrointestinal tract and can also give clues about signs of complications, like perforation or obstruction.^8^

Endoscopy is the mainstay of treatment for the removal of foreign bodies in gastrointestinal tract. Indications for endoscopic removal are presentation within 24 hours, absence of peritonitis, obstruction or perforation.^9^,^10^ According to the latest available guidelines, all sharp and large objects should be removed endoscopically after one day and there is no role of observation in these cases. Endoscopy may be attempted in cases presenting late, but failure rate is high.^10^

Surgical intervention should only be taken in case of signs of peritonitis, failure of endoscopy as repeated attempts may cause bleeding or perforation, presence of sharp objects, object size greater than 10 cm or the presence of foreign body inside gut for more than 10 days.^9^,^10^

Although laparotomy is gold standard for removal of ingested foreign body, but laparoscopy is nowadays becoming more popular as results in early recovery.^6^ In our case the position of the nail did not change even after 20 days. Laparotomy was planned as laparoscopic instruments are not available in emergency and expertise of minimal invasive surgery are lacking at junior level. Per operatively, there was a nail of 10 cm with no sharp edges (Fig.1B), embedded in the second part of duodenum leading to sealed perforation due to pressure necrosis by its head and no signs of peritonitis. In this case duodenorraphy after foreign body extraction through the same perforation is the recommended treatment.^9^

## CONCLUSION

Patients of foreign body ingestion should be monitored closely and removal should be done within a week if they are not passed spontaneously. In cases of sharp and large foreign bodies in upper gastrointestinal tract, endoscopic removal is recommended after 24 hours of ingestion.

### Author`s Contribution

**FN:** Did manuscript writing, editing and review of manuscript, final approval of manuscript & accountability.

**BQ & MSY:** Conceived, designed data collection and final approval of manuscript & accountability.
